# Mood configurations and their relationship to immune system responses: Exploring the relationship between moods, immune system responses, thyroid hormones, and social support

**DOI:** 10.1371/journal.pone.0216232

**Published:** 2019-05-31

**Authors:** Jolly Masih, Frank Belschak, J. M. I. Willem Verbeke

**Affiliations:** 1 Erasmus School of Economics, Erasmus University, Rotterdam, The Netherlands; 2 School of Economics, University of Amsterdam, Amsterdam, The Netherlands; University of Roehampton - Whitelands College, UNITED KINGDOM

## Abstract

Analyzing data on 2,057 healthy subjects in the Dutch Lifelines database we explore the relationship between immune system responses, thyroid hormone functioning and people’s mood that is expected to be moderated by social support. We focus (1) on the innate immune system cell count: monocytes, eosinophil granulocytes, basophilic granulocytes, neutrophil granulocytes; and thrombocytes; and (2) on the adaptive immune system cell count: lymphocytes (T, B and NK cells). Moods were measured on the positive (PA) and negative (NA) dimensions of the PANAS scale, divided in four groups based on their PA and NA median scores: hedonic, positive mood, negative mood and anhedonic. We focus further on (3) thyroid cells: T3 and T4; and (4) on social support. We found significant differences between mood groups and mean cell counts for basophilic granulocytes and thrombocytes but not for monocytes, eosinophil granulocytes and neutrophil granulocytes in the innate immune system. However, in the adaptive immune system we found mean lymphocyte cell counts to be different in all four mood groups. We also found that T3 and T4 levels differ significantly across all mood groups and work in very close association with lymphocytes to activate the adaptive immune system. These differences were most significant in the hedonic and anhedonic groups. The findings allow us to better understand mood groups, especially the hedonic and anhedonic groups, and open up new avenues for intervention.

## Introduction

In the field of psychoimmunology [1] or affective immunology [2], researchers have shown that immune system responses and moods are related. However, findings are not always consistent. Negative moods, for instance, are related to a higher lymphocyte cell count [3], higher basophilic granulocyte cell count [4, 5] and higher thrombocyte cell count [6, 7]. Positive moods are also related to basophilic granulocyte and thrombocyte-increased cell counts [8] and to a higher lymphocyte cell count [9]. Another study shows that the interaction between basophilic granulocyte and thrombocyte comes with high arousal of both positive and negative valence [10]. In addition, higher lymphocyte cell count is related to both anger and happiness [9, 11].

The reason behind the diverging findings about the relationship between moods and the immune system is that immune system responses and moods are multifaceted concepts. Immune system cells have binding sites for hormones such as thyroid hormone (TH) that affect mood. Immune system responses and moods also have the potential to cross talk to one another [12]. All the different cross talks between immune system cells and moods reflect homeostatic processes, which are fundamental mechanisms to maintain a healthy state of both mind and body [13].

In this paper, we first make a distinction between the innate immune system cells and adaptive immune system cells and consider whether both might have different relationships with moods [14]. Hence, we study innate and adaptive immune system levels separately. Second, “mood” is an umbrella concept that reflects various processes (ranging from stress, anxiety, optimism and depression) and dimensions (valence and intensity). Here we focus on positive and negative moods that reflect a temporary state of mind and are last longer than, for instance, emotions that are immediate responses to a specific event [15]. While most studies focus on people’s scores for negative moods rather than positive moods [2, 12], we study the relationship between immune system levels and both positive and negative moods and create mood configurations, thus forming mood groups based on these configurations. For instance, based on their positive and negative mood scores we categorize people in high-high versus low-low groups labeled as the hedonic group versus the anhedonic group.

Third, because the adaptive immune system level in particular has TH binding sites, the TH upregulates catecholamines like serotonin and epinephrine which affect moods [16, 17, 18]. Thus, we study whether the TH (triiodothyronine or T3 and thyroxine or T4) part of the adaptive immune system level is associated with mood [19]. In addition, adaptive immune system levels and TH functioning are social-context sensitive, meaning that TH affects the sympathetic nervous system (like epinephrine) that is known to be affected by social environmental factors such as stress or loneliness [18]. Concretely, we study whether the immune system level and thyroid relationship in the different mood groups is moderated by social support. Such a holistic perspective, [20] for instance, allows us to better understand how moods and immune system levels are related and thus contribute to the nascent field of affective immunology [2].

## Theory

In order to study people’s moods we use the positive (PA) and negative (NA) affect schedule scale (PANAS), a self-report instrument [21] widely used in the field of psychology for both clinical and non-clinical populations. Moods function like emotions but unlike emotions, they are “not necessarily directed at anything” [22], yet they are consciously felt and always present in the background. Put concretely, we explore whether people’s PA and NA moods might be related to the immune system level cell count.

**Fig 1:**
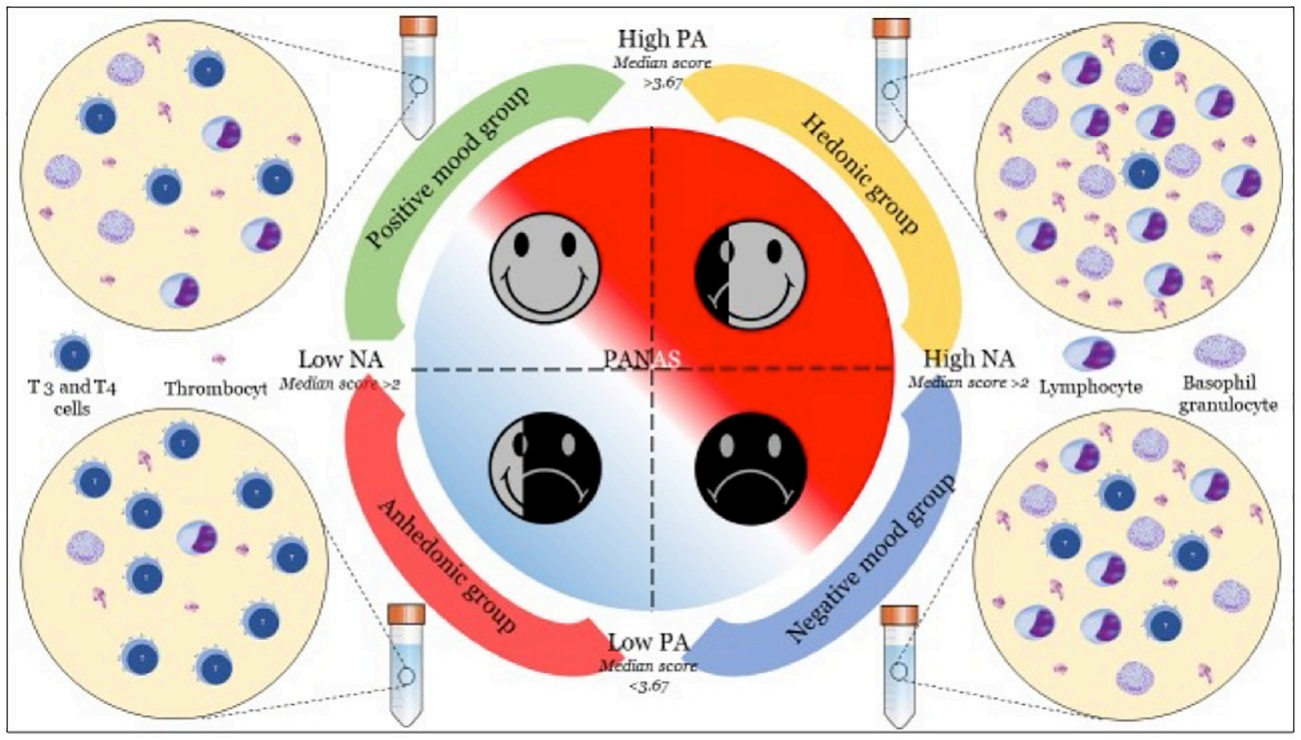
Configuration of mood groups. Four mood groups namely hedonic group, negative mood group, positive mood group and anhedonic group were created based on the median scores, for PA = 3.67 and NA = 2.00.

Using epidemiological data from 2,057 subjects in the Lifelines database in the Netherlands, we created four different mood configurations based on the median score [21] of the positive (PA) and negative (NA) dimensions of the PANAS scale: (1) the hedonic group with high scores on both PA and NA; (2) the negative mood group with a low score on PA but high on NA; (3) the positive mood group with a high score on PA but low on NA; and (4) the anhedonic group with a low score on both PA and NA. A salient feature of this study is that the anhedonic group is prone to developing depression [23] and is characterized as having blunted affective levels as well as both low PA and NA mood states [19]. We conceive the hedonic group as people whose constantly oscillating high PA and NA moods might indicate highly sensitive affective levels to social environmental changes [23]. As shown in Fig 1, note that mood groups and immune system cells are mutually connected and cross talk with one another, which makes predicting the direction of relationship very difficult [24].

Next, in order to study the relationship between immune system level cell count and moods more closely, we differentiate between the innate and adaptive immune systems as they function differently. Innate immune system cells (thrombocytes and basophilic granulocytes) initiate early inflammation by triggering histamine and serotonin, which are associated with both positive and negative moods [8, 25, 26]. Innate immune system cells (basophilic granulocytes via histamine) alert NK cells (lymphocytes), which build rapidly at the site of inflammation [27]. In this process, TH secretion (T3 and T4) comes into play as the lymphocytes possess binding sites for these hormones and are affected by people’s moods. TH also affects the pathological activity of lymphocytes [19, 28]. As mentioned previously, lymphocytes-thyroid functioning is related to moods and affected by social support dynamics [29]. Researchers have noted that TH (T3 and T4) secretion levels are associated with mood alterations and such disorders as bipolar disorder, depression, hedonia and anhedonia [24].

These mechanisms motivated us to explore the differential effects of the mood groups on TH secretion levels (T3 and T4 cells), which are sensitive to stress and social environment and also affect lymphocyte (NK, T and B) cell count [17, 28]. We particularly expect the relation between TH secretion levels and lymphocyte cell count to occur in the anhedonic group because hyposecretion of T3 and T4 leads to hypothyroidism and depression [30, 31]. Hypothyroidism is known to be related to lower NK cell count, which is a sign of immune system malfunctioning in people suffering from anhedonia [31]. In contrast, people who suffer from hyperthyroidism experience mood swings; hence we expect to see T3 and T4 hypersecretion in the hedonic group [31]. TH hypersecretion is known to increase NK cell count in the hedonic group [32].

**Fig 2:**
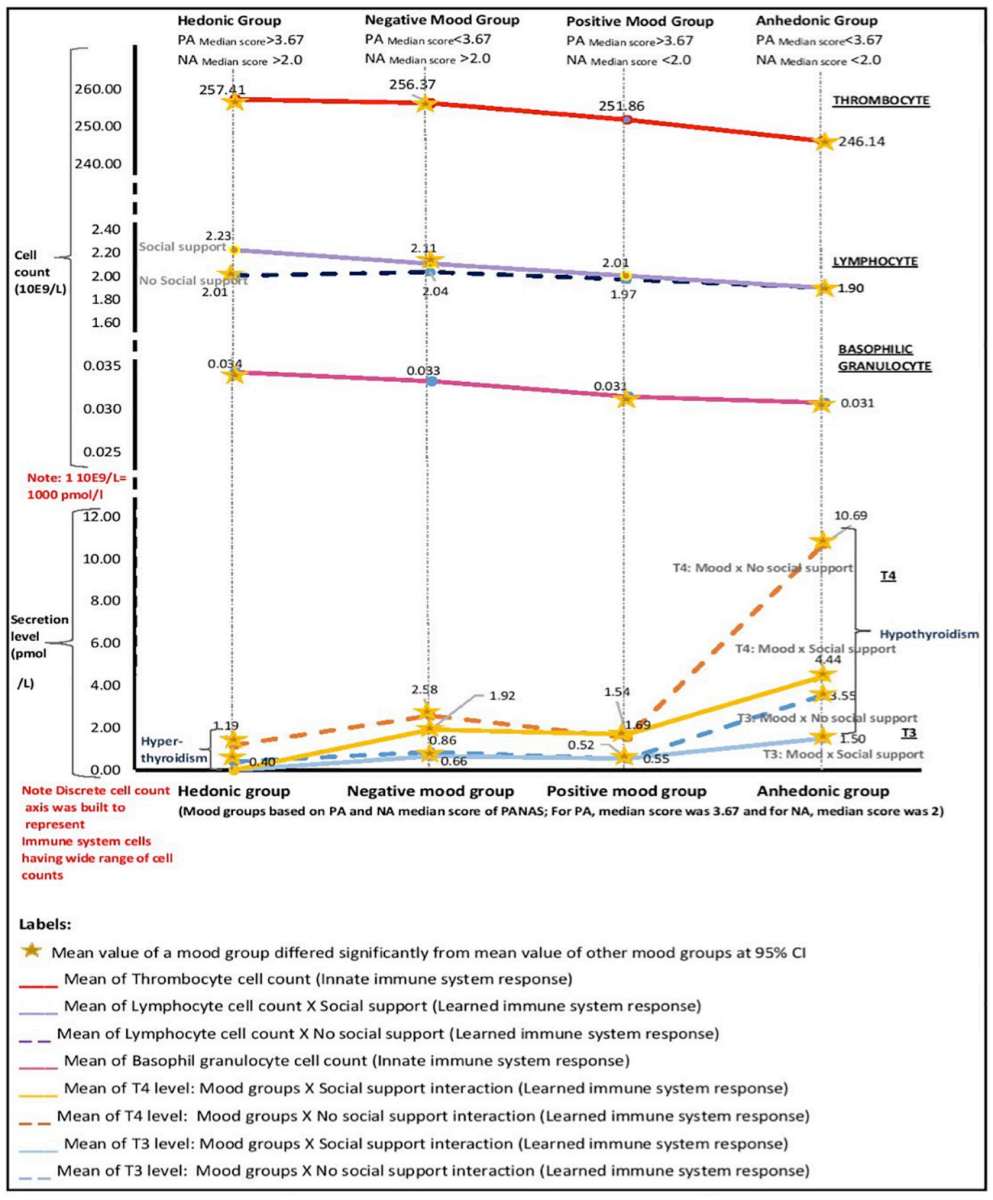
Immune system responses and mood groups interaction. Multivariate analyses of variance (MANOVA) was conducted with the mood groups as the independent variable and the immune system responses as dependent variables. Monocytes and eosinophil granulocytes were not found significant in the MANOVA test and are thus not included in the figure.

Importantly, both the adaptive immune system and thyroid functioning are sensitive to social-context feelings, such as loneliness, and social isolation or social support. First, loneliness reduces the NK cell count, disturbs the ratio of helper T cells and suppressor/regulatory T cells leading to decreased lymphocyte proliferation [33, 34]. Second, loneliness affects moods, creating disturbances in TH functioning leading to hypersecretion or hyposecretion of T3 and T4 [34]. As shown in Fig 2, hence, we expect to see the relationship between TH secretion levels and the adaptive immune system level cell count to be moderated by social support and mood group interaction.

## Method

### Data source

Data were collected from the Lifelines Cohort Study biobank in Groningen, the Netherlands [35]. This large-scale collection makes biological data and samples available for epidemiological research, following a standard application procedure. (Coded under Proposal no. OV16_0366–https://www.lifelines.nl/–this study was granted permission to analyze the Lifelines data by the ERIM ethics commission of the Erasmus University, Rotterdam under number2016/02/11-05483wve). Every five years, participants visit one of the Lifelines sites in the north of the Netherlands for a physical examination.

### Sample size

We selected the adult group, one segment of the Lifelines database, comprising 2,057 participants (18–65 years of age) and study moods (based on the PANAS scale), immune system level cell count, TH functioning and social support levels.

### Demographic information

The population consisted of 893 (43.4%) females and 1164 (53.6%) males. All respondent were Caucasians and their ages ranged between 18 and 65 years (mean = 44.61, *SD* = 8.26)

### PANAS scale

This was chosen as the scale of interest from the Lifelines database [36, 37]. The PANAS scale covers a series of items (ten for each dimension), posing questions like “how often have you felt nervous in the past four weeks.” The scale includes items which respondents need to answer on a 1 (rarely) to 5 (very often) scale: for PA (including the variables inspired, enthusiastic, alert, attentive, interested, strong, determined, active, proud and satisfied); and for NA (including the variables nervous, jittery, scared, afraid, upset, guilty, ashamed, distressed, irritable and hostile). The PA and NA dimensions were obtained after performing factor analysis on the PANAS scale. Cronbach’s alphas for both PA and NA dimensions were α = 0.77 and α = 0.86 respectively. Based on the median scores, for PA = 3.67 and NA = 2.00, we created four configurations for PA and NA combinations, thus placing the subjects in four groups.

#### Hedonic group

(High Positive and High Negative Score): Individuals who scored higher than the median on both PA (score>3.67) and NA (score>2.0) (N = 593).

#### Negative mood group

(Low Positive and High Negative Score): Individuals who scored lower than the median on PA (score<3.67) and higher than the median on NA (score>2.0) (N = 482).

#### Positive mood group

(High Positive and Low Negative Score): Individuals who scored higher than the median on PA (score>3.67) and lower than the median on NA (score<2.0) (N = 717).

#### Anhedonic group

(Low Positive and Low Negative Score): Individuals who scored lower than median score on both PA (score<3.67) and NA (score<2.0) (N = 265).

### Social support

The Social Production Function Instrument for Level of Well-Being Scale (SPF-IL Scale) [38] contains nine items (e.g., “Don’t you pay attention to yourself?”). A composite was formed out of these nine items and those lower than 1.5 were coded as “1” (“lonely group” or “no social support group”) and those higher than 1.5 were coded as “2” (“not lonely group” or “group having social support”) [39].

### Biomarkers extracted from blood

All participants were asked to be sober for blood collection, which is this case was no food after 24:00 hours.

#### Immune system cells

For the innate immune system cells, thrombocytes, monocytes, basophilic granulocytes, neutrophil granulocytes and eosinophil granulocytes were selected for this research (see Table 1). Unit of count immunity variables was 10E9/L. For the adaptive immune system, the lymphocytes are selected. The ratio of lymphocytes cell composition is as follows: CD3 (T cell 1–3%), CD4 (T suppressor/regulatory cell 0.5–1.3%), CD8 (T suppressor/regulatory cell 0.3–0.8%), CD19 (B cell 0.2–0.5%) and CD16/56 (NK cells 0.1–0.7%). The duration of collection of these cells is 12 years (Diagnostic Kompas 2003 as provided by https://www.lifelines.nl/; [40]). Please note that we took the exact cell count data for the immune system cells and not the percentage, since we were interested in an accurate quantity of cell count levels to study the effect of immune system cells on the four mood groups.

**Table 1:** The immune system parameters.

Label	Name	Type	Element no.	Start (period)	End (period)	Tube	Kit name	Supplier name	Supplier country
Basophilic Granulocytes (10E9/L)	BA	Blood	784	27-11-2006	1-1-2018	EDTA Sysmex	XE2100—System	Sysmex	Japan
Eosinophil Granulocytes (10E9/L)	EO	Blood	783	27-11-2006	1-1-2018	EDTA Sysmex	XE2100—System	Sysmex	Japan
Free T3 (pmol/L) (triiodothy-ronine)	FT3	Blood	10743	16-11-2009	15-10-2011	Heparin	Elecsys free triiodothy-ronine (ECLIA (electro-chemilum-inescence immune-assay), competition principle)	Roche (Modular E)	Germany
Free T4 (pmol/L) (thyroxine)	FT4	Blood	10744	16-11-2009	15-10-2011	Heparin	Elecsys free thyroxine (ECLIA (electro-chemilum-inescence immune-assay), competition principle)	Roche (Modular E)	Germany
Lymphocytes (10E9/L)	LY	Blood	781	27-11-2006	1-1-2018	EDTA Sysmex	XE2100—System	Sysmex	Japan
Monocytes (10E9/L)	MO	Blood	782	27-11-2006	1-1-2018	EDTA Sysmex	XE2100—System	Sysmex	Japan
Neutrophil Granulocytes (10E9/L)	GR	Blood	780	27-11-2006	1-1-2018	EDTA Sysmex	XE2100—System	Sysmex	Japan
Thrombocytes (10E9/L)	TR	Blood	771	27-11-2006	1-1-2018	EDTA Sysmex	XE2100—System	Sysmex	Japan

#### TH secretion levels

T3 and T4 secretion levels were selected for this study. Unit of count was pmol/L (see Table 1). The secretion of T3 and T4 were measured with Elecsys free triiodothyronine (ECLIA, electrochemiluminescence immunoassay), (Roch, Germany) [40].

### Research tool

Multivariate analysis of variance (MANOVA) was used where immune system level cell counts were taken as dependent variables and mood groups (based on PANAS scale) as independent variables. Confidence interval was taken as 95% and basic assumptions such as independence of observations, dependent variable on interval measurement, dependent variables multivariate normally distributed and equal population covariance matrices of each group were followed.

Note that we took mood groups as an independent variable to facilitate statistical computations. In reality, mood groups and immune system level cell counts are closely associated and cross talk to one another. Thus we do not predict or state any specific direction of this relationship.

## Results

To test whether the mood groups and immune system level cell counts differed significantly, we computed multivariate analyses of variance (MANOVA) with the mood groups as the independent variables and the immune system level cell counts as dependent variables. Fig 1 displays the different mood group configurations and Fig 2 displays the interaction between the immune system level cell counts and the different mood groups.

First, we calculated MANOVA for the mood groups and innate immune system levels (including cell count of basophilic granulocytes, eosinophil granulocytes, monocytes and thrombocytes). The results showed that the mood groups displayed significantly different immune cell counts for basophilic granulocytes (*F* = 3.005, *p* = .029, η^2^ = .004) and thrombocytes (*F* = 2.637, *p* = .048, η^2^ = .004), but not for monocytes (*F* = 2.194, *p* = .087, η^2^ = .003) or for eosinophil granulocytes (*F* = 2.362, *p* = .070, η^2^ = .003) or neutrophil granulocytes (*F* = 1.10, *p* = .309, η^2^ = .0). Table 2 (upper part) shows the mean values of the innate immune system level cell counts for the four mood groups. Figure A and Figure B in S1 File shows the plots of mean basophilic granulocytes and thrombocytes respectively on different mood groups, since only these two innate immune system cells showed a significant difference in mean distribution across all four mood groups.

**Table 2:** Means and standard deviations (in parentheses) of immunity system cells for the four mood groups.

	PA high–NA high	PA high–NA low	PA low–NA high	PA low–NA low
Monocytes	.497 (.166)	.475 (.167)	.494 (.171)	.488 (.186)
Basophilic granulocytes	.034 (.021)	.031 (.019)	.033 (.021)	.031 (.022)
Eosinophil granulocytes	.200 (.130)	.180 (.127)	.182 (.125)	.173 (.132)
Neutrophil granulocytes	3.44 (1.27)	3.33 (1.31)	3.47 (1.39)	3.39 (1.53)
Thrombocytes	257.410 (57.191)	251.862 (62.160)	256.373 (62.366)	246.140 (61.346)
Lymphocytes	2.006 (.628)	1.987 (.647)	2.074 (.723)	1.966 (.657)
T3 cells	.401 (1.427)	.532 (1.582)	.758 (1.947)	1.780 (2.518)
T4 cells	1.183 (4.222)	1.604 (4.781)	2.243 (5.646)	5.286 (7.492)

Table 3 shows the MANOVA results for the innate immunity cell count. Results of post-hoc tests further specified that for thrombocytes, the anhedonic group scored significantly lower than both the hedonic group (mean difference = -11.270, *s*.*e*. = 4.486, *p* = .012) and the negative mood group (mean difference = -10.234, *s*.*e*. = 4.643, *p* = .028). For basophilic granulocytes, the hedonic group scored significantly higher than both the anhedonic group (mean difference = .004, *s*.*e*. = .002, *p* = .019) and the positive mood group (mean difference = .003, *s*.*e*. = .001, *p* = .012). Based on mean values, the results indicate that the anhedonic group has the lowest innate immune system level cell counts in contrast to the hedonic group which has the highest innate immune system level cell counts (see Tables 2 and 3).

**Table 3:** Results of MANOVA for innate immunity cell counts.

	*Basophilic granulocytes*			*Thrombocytes*			*Eosinophil granulocytes*			*Monocytes*			*Neutrophil*		
	F-value	p-value	η^2^	F-value	p-value	η^2^	F-value	p-value	η^2^	F-value	p-value	η^2^	F-value	p-value	η^2^
PANAS	3.005	.029	.004	2.637	.048	.004	2.362	.070	.003	2.194	.087	.003	1.19	.309	.0

Next, we tested the interactive effect of mood groups and social support on the adaptive immune system level cell counts. Here, we computed MANOVA using the mood groups, social support, and the interaction of these two variables as independent variables and the adaptive immune system levels (including cell count of lymphocytes, T3 level, and T4 level) as dependent variables. The mean values of the adaptive immune system level cell counts for the four mood groups can be found in Table 2 (lower part). For better understanding, Figure C, Figure D and Figure E in S1 File show the mean counts for lymphocyte cells, and T3 and T4 levels respectively on different mood groups, since all showed significant differences in mean distribution across all four mood groups. The interaction between the mood groups and social support had a significant effect on all three adaptive immune system cells (lymphocytes: *F* = .164, *p* = .042, η^2^ = .000; T3 level: *F* = 12.305, *p* = .000, η^2^ = .018; T4 level: *F* = 13.285, *p* = .000, η^2^ = .019). Tables 4 and 5 present the MANOVA results. As shown in Figs 1 and 2, Tables 4 and 5, the mean values of the adaptive immune system level cell counts for the different configurations of the mood groups and social support (also see Figure C, Figure D and Figure E in S1 File).

**Table 4:** Means and standard deviations (in parentheses) of adaptive immunity cell counts for the mood groups x social support interaction.

Social support	PANAS	Lymphocytes	T3 cells	T4 cells
No	PA high–NA high	2.005 (.629)	.403 (1.431)	1.189 (4.232)
	PA high–NA low	1.974 (.657)	.521 (1.573)	1.539 (4.659)
	PA low–NA high	2.048 (.752)	.863 (1.996)	2.577 (5.947)
	PA low–NA low	1.903 (.678)	3.553 (2.617)	10.686 (7.916)
Yes	PA high–NA high	2.227 (.356)	.000 (.000)	.000 (.000)
	PA high–NA low	2.006 (.633)	.548 (1.596)	1.694 (4.954)
	PA low–NA high	2.109 (.693)	.657 (1.898)	1.922 (5.333)

**Table 5:** Results of MANOVA for adaptive immunity cell counts.

	*Lymphocytes*			*T3 cells*			*T4 cells*		
	F-value	p-value	η^2^	F-value	p-value	η^2^	F-value	p-value	η^2^
PANAS	2.229	.063	.003	45.705	.000	.063	46.428	.000	.064
Social support	.949	.030	.000	5.860	.016	.003	6.069	.014	.003
PANAS x social support	.164	.042	.001	12.305	.000	.018	13.285	.000	.019

Tables 4 and 5 and Figure C, Figure D and Figure E in S1 File show that the highest mean lymphocyte cell count occurred in the hedonic group who received social support. However, post-hoc tests indicated that the mean differences between this group and the other groups were not significant (mean differences ranged from .117 to .324, all *p*>.10). Similarly, individuals who received no social support did not differ substantially in terms of lymphocyte cell count levels across the mood groups except for the negative mood group with social support that showed significantly higher mean scores for lymphocyte cell count than the hedonic group with no social support (mean difference = -0.104, *s*.*e*. = .050, *p* = .038), the positive mood group with no social support (mean difference = .136, *s*.*e*. = .053, *p* = .011) and the anhedonic group with social support (mean difference = .133, *s*.*e*. = .061, *p* = .029). This indicates that when people with negative mood get social support, their lymphocyte cell count increases rapidly.

For T3 and T4 levels we found a different picture. Here, highest mean levels of adaptive immune system level cell counts were reached in the anhedonic group. While this holds both for groups that received and did not receive social support, those without social support showed the highest mean levels of T3 and T4 of all mood groups with social support combinations. Post-hoc tests confirmed the finding that both mean T3 and mean T4 levels of the anhedonic group receiving no social support were significantly higher than the mean T3 and T4 levels of any other group (T3 level: mean differences ranged from 2.051 to 3.553, all *p* < .01; T4 level: mean differences ranged from 6.249 to 10.868, all *p* < .01), including the anhedonic group who received social support (T3 level: mean difference = 2.51, *s*.*e*. = .316, *p* = .000; T4 level: mean difference = 6.249, *s*.*e*. = .936, *p* = .000). The anhedonic group with social support showed significantly higher mean T3 and T4 levels than the other mood groups with social support (T3 level: mean differences ranged from .844 to 1.502, all *p* < .01; T4 level: mean differences ranged from 2.514 to 4.437, all *p* < .01) (see Tables 4 and 5).

Although we tried gender as a co-variate for both innate and adaptive immune system level cell counts, we found no significant differences in the means. Thus we assume that males and females respond similarly in immune system levels across the four mood groups.

## Discussion

In this paper we hypothesized about the relationships between mood groups and innate and adaptive immune system level cell counts. We suggested that the adaptive immune system inducing lymphocyte-thyroid functioning might be affected by the interaction between mood groups and social environment [2].

Both immune system levels and moods are known to cross talk with one another [2]. Given that thyroid functioning and adaptive immune system level cell counts [17, 34] also cross talk potentially and thyroid function is affected by moods [30], this study allows a closer look at these mutual relationships.

First, we hypothesized that the innate immune system level cell counts—thrombocytes, monocytes, basophilic granulocytes, neutrophil granulocytes and eosinophil granulocytes—would be different across all four mood groups. However, only the basophilic granulocyte and thrombocyte cell counts showed significant differences in the four mood groups; the monocytes, neutrophil granulocytes and eosinophil granulocytes cell counts were not significantly different. Note that basophilic granulocytes and thrombocytes cell counts were highest in the hedonic group and lowest in the anhedonic group. The negative and positive mood groups scored somewhere in the middle.

With regard to mood group-innate immunity level relationships, basophilic granulocytes produce histamine and serotonin, which are responsible for early inflammation when deviation from homeostatic state is detected [41]. These physiological conditions confirm a growing literature that finds that this chain reaction affects positive and negative moods in people [25, 26, 42]. Note that this finding concurs partially with the findings of our study, where the hedonic group scores high on both PA and NA.

Second, similar patterns were found for the thrombocytes, also called as platelets. Here again the hedonic group scored the highest, the anhedonic the lowest and the negative and positive group somewhere in the middle. The main function of thrombocytes is to contribute to homeostasis and the process of stopping on-site bleeding in the endothelium, building up rapidly at the inflammation site [43]. Thrombocytes are reported to produce mood-modulating serotonin. Thrombocytes and basophilic granulocytes cell counts are known to be associated with positive and negative moods via histamine and serotonin interactions [8, 44]. In addition, thrombocyte cell count is also found to be related to mood disorders such as bipolar affective disorder [6, 45].

Moving on to the functioning of adapted immune system level cell counts, we found differential patterns compared to the innate immune system cell counts. First, all mood groups (hedonic, positive, negative and anhedonic) were found to be associated with adaptive immune system level cell counts, which substantiates earlier work by a few scientists [8, 29]. It was found [46] that negative emotions, such as bad mood, depression and anxiety augment the production of IL-6 (found in NK cells) and produce inflammatory level, thereby producing heightened responsiveness to subsequent stressful events. It was found that hedonic group experiences rapidly increase in lymphocyte cell counts in the event of happiness and anger of mood swings [11].

We also found that the TH (T3 and T4) secretion levels in the four mood groups had precisely the opposite pattern (mirror reflection) of the adaptive immune system cells or lymphocytes, thus indicating that they are closely related. Specifically, the literature shows that the anhedonic group might be more prone to hypothyroidism, and the hedonic group might be more prone to hyperthyroidism [17, 30, 31]. Again, the negative and positive mood groups had TH (T3 and T4) secretion somewhere in the middle. Most importantly, this relationship occurred only when social support was associated as a moderator of this homeostatic process. That is, when people scored low on social support, we observed more fluctuation in TH secretion. This might also indicate that when people receive social support, they have relatively stable TH secretion levels. Note that similar observations have been made in the literature, indicating that TH secretion and immune system levels are related to moods.

With regard to mood disorders and TH functioning, a variety of thyroid abnormalities has been identified in patients with such mood disorders as depression and bipolar disorder [24]. TH on lymphocytes frequently affects immune system level alterations in physiological and pathological activity [19]. Hyper- and hypothyroid functioning is found to be a major cause of both hedonia and anhedonia [31]. Another study found hypothyroidism a major cause of chronic depression, an all-time low mood state and low lymphocyte cell count [30]. In contrast, people with hyperthyroidism are found to be emotionally reactive and impulsive, which mimics the hedonic group in this study. Note that the hedonic group was found to have a comparatively high number of lymphocytes [31]. The lymphocyte and TH secretion interaction was also found to be moderated by social support [29].

The bottom line of this study is that different mood group trigger distinct changes in innate immune system level cell counts, especially basophilic granulocytes and thrombocytes in the hedonic and anhedonic groups. We found this relationship to be direct and unaffected or moderated by TH, social support, or gender. Next, the adaptive immune system level cell count and TH secretion is associated with moods and social environment dynamics. Indeed, most studies of the relationship between immune system cells and the social environment have targeted adaptive immune system level cell counts and less so innate immune system level cell counts [47, 48]. Note again that immune system cells and moods cross talk potentially which makes it hard to conclude a causal relationship [2].

In order to study moods, we categorized the groups according to the participant’s overall score configuration and not on their score for only one scale, PA and/or NA, for example. One question we could pose is, what else can we learn about the hedonic and anhedonic groups? First, we are aware that both ‘hedonic’ and ‘anhedonic’ are metaphors, indicating the participant’s scores on PA and NA, respectively. We could have labeled the hedonics as the impetuous, impulsive or spontaneous group. In searching the literature we have been unable to find any studies that properly label this group. Our study on moods and immune system level cell counts, however, was inspired by such researchers [2], among others, who observed that “surprisingly, very little has been published on the effect that positive emotional states have on immune system levels” [12]. Here we extend this question by studying not only how high versus low PA or NA moods relate to immune system levels, but also what we can learn about the PA and NA configurations that formed the basis of our mood groups. Here follow a few conjectures.

The anhedonic group is most affected by social context, specifically an unsupportive social environment. Earlier research has indicated that lonely people show a lower lymphocyte cell count [46]. To strengthen our results we undertook a small thought-step, comparing this anhedonic group with people suffering from depression. Some cytokines are known to activate brain serotonergic systems, which affect depression and its treatment [49]. It was found that inhibiting pro-inflammatory cytokines or their signaling pathways could improve depressed mood [50]. The anhedonic group could perhaps be treated with antidepressant drugs like serotonin-specific reuptake inhibitors (SSRI) [51, 52]. Next, Coyne [53] has noted that depressive people trigger a negative or separation level in other people. Depressive moods are emotionally contagious and affect those people who interact with sufferers of depression [54]. Perhaps the anhedonic group studied here, who did not score high on either positive or negative mood, might establish their own feelings of isolation and low social support. This feeling of isolation might in turn make these people sensitive to stress due to hypothyroidism.

Similarly, the hedonic group is also affected by a positive social environment and this perhaps explains why these people have mood swings: when social support is high they feel enjoyment, thus high PA. But equally, when social support is low they might experience high NA. This confirms to some extent the conjecture by Segerstrom [16] who suggests that optimists might suffer from sudden changes when a task becomes challenging, which might affect their immune system level cell counts. Again, this is simply conjecture but it could help us understand this mood group better. Please note, according to the results of our study, people high on social support are less likely to have mood swings and their moods and immune system interactions are more stable.

These observations, specifically about the two extreme groups, could allow fellow researchers to draw translational implications. For instance, could therapists help the anhedonic group overcome their mood state by affecting their immune system levels or thyroid functioning, which all cross talk to one another? An initial step could be to help these people develop enriching social contacts which, in turn, would make them less prone to social isolation and social stress. Similarly, some researchers suggest that immunotherapy would help, by encouraging people to exercise or increase physical activity, for example, or by affecting the gut-brain axis functioning, or by helping people with sleep problems to improve hygiene or refrain from consuming unhealthy food and alcohol [12, 55]. On the one hand, cognitive behavioral therapy could help this mood group to change their emotional tone that then affects their immune system levels. On the other hand, therapies could focus on thyroid function and prescribe medication or add iodine to the diet for people suffering hypothyroidism [56].

Besides the topics mentioned above, biochemical parameters, such as serum cytokine concentrations, are also suggested to have an important role in the development of mood disorders [57]. Pro-inflammatory cytokines are found to be elevated in patients with anxiety and depression [58]. It is expected that insufficient action of TGF-β1 (immunoregulatory cytokine that has a multitude of suppressive actions on T cells, B cells, and macrophages) would lead to the increased inflammation described in depressive disorders [59].

Mao R et al. reported that two classes of mood disorders (body dysmorphic disorder (BDD) and major depressive disorder (MDD)) may present different inflammatory features, which are affected by medication [60].

Recent evidence suggests that stress hormones may actually facilitate inflammation followed by depression, through induction of various cytokines including IL-1, IL-6, IL-8, IL-18 and TNF (tumor necrosis factor). Thus, a dysfunctional neuroendocrine-immune interface may play a role in the pathogenesis of depression, and its complications [61].

Finally, there are many questions to be asked about the relationship between people’s mood and immune system levels. These questions include what is the nature of immune cells, inflammatory markers such as cytokines and the chemokines involved, the position of inflammation in the sequence of events leading to mood disorders, whether length of exposure to thyroid hormones (T3 and T4) is important, and what is the exact mechanism behind the cross talk between immune system (innate and adaptive) and hypothalamic-pituitary-adrenal (HPA) axis. Investigating these questions will help us to clarify the biological mechanisms between mood and immune system [62]. Another interesting question that researchers may explore further is our finding that the relationship between immune system levels and mood groups is not affected by gender.

## Study limitations

The relationships we found are significant but their adjusted R^2^ is relatively low. However, this kind of result is common in the epidemiological literature. We did not perform multiple comparison corrections since we were looking at the effect of different immune system level cell counts. When they function together, like a unit or team, they trigger a series of chain reactions as system-based processes and biologically speaking are not independent of each other. This could be perceived as a study limitation. Please also note that the Lifelines cohort data used deals with a healthy population and thus any differences based on, say, a comparison of the healthy and unhealthy would be minor. In addition, the epidemiological—not experimental—condition of this study might explain the low correlations found.

As this was an epidemiological study we did not include exact neuro-endocrine and immune system pathways but chose to build on existing theoretical frameworks developed by eminent researchers [12, 34].

## Supporting information

S1 File Supporting figures. (DOCX)
