# Supplementary material for: Mood configurations and their relationship to immune system responses: Exploring the relationship between moods, immune system responses, thyroid hormones, and social support
Source: PLoS One. 2019 May 31;14(5):e0216232. doi: 10.1371/journal.pone.0216232 (PMC6544341; doi:10.1371/journal.pone.0216232)
Supplement: S1 File — (DOCX) [file pone.0216232.s001.docx]

**Supporting material**

List of Figures

Figure A: Mean basophilic granulocytes cell counts on mood groups

Figure B: Mean thrombocytes cell counts on mood groups

Figure C: Mean lymphocytes cell counts for mood groups x social support interaction

Figure D: Mean T3 levels for mood groups x social support interaction

Figure E: Mean T4 levels for mood groups x social support interaction

Note: In hedonic group x social support interaction of T3 and T4 cells, no observations were recorded, thus the value is zero.
